# Metabolic reprogramming by Dichloroacetic acid potentiates photodynamic therapy of human breast adenocarcinoma MCF-7 cells

**DOI:** 10.1371/journal.pone.0206182

**Published:** 2018-10-23

**Authors:** Zeiyad Alkarakooly, Qudes A. Al-Anbaky, Krishnaswamy Kannan, Nawab Ali

**Affiliations:** 1 Department of Biology, College of Science, University of Diyala, Diyala, Iraq; 2 Department of Biology, College of Arts, Letters and Sciences, University of Arkansas at Little Rock, Little Rock, Arkansas, United States of America; University of South Alabama Mitchell Cancer Institute, UNITED STATES

## Abstract

Aberrant glycolytic metabolism is one of the hallmarks of carcinogenesis and therefore reversal of metabolic transformation is a promising drug target in cancer treatment strategies. Dichloroacetic acid (DCA) is known to target the glycolytic pathway in cancer cells and facilitates reversal of metabolic transformation from aerobic cytosolic accumulation of pyruvic acid, “the Warburg effect”, to mitochondrial oxidative phosphorylation. Recently, combination therapy particularly involving photodynamic therapy (PDT) has received considerable attention in oncology. We hypothesized that if DCA and PDT are combined, they might potentiate mitochondrial dysfunction and induce apoptosis by a reactive oxygen species (ROS) dependent pathway. We used MCF-7 cells as our *in vitro* model and 5-aminolevulinic acid (5-ALA) dependent PDT therapy to test our hypothesis. We found that combinatorial treatment of MCF-7 cells with PDT and DCA not only increased cell growth inhibition, but also affected mitochondrial membrane integrity perhaps via production of ROS, and enhanced apoptosis. Further, our results on ATP release during the combined treatment demonstrate that immunogenic cell death (ICD) is likely to be a potential mechanism by which PDT and DCA induce cancer cell death. Taken together, our study suggests a novel way of sensitizing MCF-7 cells for accelerated induction of apoptosis and ICD in these cells. The findings included in this study might have direct relevance in breast cancer treatment strategies.

## Introduction

Breast cancer (BC) is a major health issue worldwide. It is estimated that 1.38 million women are diagnosed with BC annually [1–3]. Surgery and radiation are the two major conventional therapies used for disease control at the local level, whereas chemotherapies are used to control metastatic disease [4]. In spite of these advancements, the metastatic BC remains an incurable disease for the majority of patients due to therapy-resistance and relapse [5]. In recent years, combination therapies involving radiotherapy, immunotherapy and chemotherapy have proven to be more effective in the control of aggressive cancers including melanoma, lung cancer and leukemia [6–8].

The seminal work by Craig Thompson and colleagues has demonstrated that metabolic traits of tumor cells are crucial for tumor survival under conditions of hypoxia and limited nutrient availability [9]. Unlike normal cells, cancer cells primarily rely on aerobic glycolysis to generate energy needed for various cellular processes and this phenomenon is termed as “the Warburg effect” [10, 11]. The discovery of the Warburg effect has enhanced our understanding of metabolic transformation and several oncogenic signaling pathways including PI3K/AKT/mTOR, p53, AMPK and others [12]. Taken together, the metabolic transformation in tumor cells is an important hallmark of oncogenesis and important therapeutic intervention target in many cancers including BC [10, 13, 14]. To this end, Golding et al (2013) used glycolysis inhibitors 2-deoxyglucose or lonidamine, taking advantage of increased aerobic glycolysis in tumor cells and combined them with 5-aminolevulinic acid (5-ALA) based PDT to achieve cytotoxicity in human breast cancer MCF-7 cells as compared to normal cells [15]. They also demonstrated that PDT was effective only when the glycolysis inhibitors were used after 5-ALA treatment.

Dichloroacetate (DCA), a small molecule of 150 Da, is a metabolic modulator that has been used in the treatment of lactic acidosis and hereditary mitochondrial diseases [16, 17]. At the cellular level, DCA acts as a mitochondria-targeting drug and is known to increase the activity of pyruvate dehydrogenase (PDH), thus resulting in a shift of pyruvate metabolism away from lactic acid formation, towards mitochondrial respiration [16]. These biochemical reactions also accelerate mitochondrial dysfunction and promote pro-apoptotic JNK signaling and subsequently induce cell death in several tumor models [16, 18, 19].

Many of the therapies used in oncology induce apoptosis in cancer cells and thus reduce the overall tumor volume and burden [20, 21]. Thus, the overall efficacy of chemotherapies is assessed by their ability to drive cytotoxicity in cancer cells. In 1994, Polly Matzinger proposed ‘danger theory’ which states that host immune system can distinguish between dangerous and innocuous endogenous signals. This observation was also extended to apoptotic cell death later on [22, 23]. The possibility that drug treatments (anthracyclines, oxaliplatin) and radiation therapy can not only exert direct cytotoxicity but also result in enhanced anti-tumor immunity of the host was attractive to immunologists and oncologists. This opened up an entirely new field of research on danger molecules that are now classified as damage-associated molecular patterns (DAMPs) [24]. Accordingly, the immune response to three molecular determinants including ATP, endoplasmic reticulum (ER) chaperon calreticulin (CRT), and the nuclear protein HMGB1 are now characterized as “immunogenic cell death (ICD)” [21, 23]. These determinants are also widely used as biomarkers of ICD [22, 23].

Recently, Garg et al [24] and others have advocated ICD as a cornerstone of therapy-induced anti-tumor immunity. Garg et al [25] has described the validity of Photodynamic Therapy (PDT) in cancer therapy which combines radiotherapy and ICD. In principle, PDT combines visible or near-infrared light with a photosensitizer to generate reactive oxygen species (ROS), which is known to efficiently kill cancer cells and increase tumor-specific antigen presentation to T lymphocytes [6, 21, 26, 27]. Thus, radiotherapy not only exerts direct cytotoxic effects on tumor cells, but also reprograms the tumor microenvironment to exert a potent antitumor immune response [28–30]. However, PDT has limited value in treating deep-seated tumors because visible light can only penetrate to a shallow depth of less than 1 cm in tissues [31]. Taken together, these studies suggest that the combinatorial therapy using PDT might be a promising strategy to efficiently kill breast cancer cells.

The present study was undertaken to investigate the combinatorial effects of DCA and PDT on the cell death mechanisms in human breast adenocarcinoma (MCF7) cells. To induce PDT, we used He-Ne laser as a light source and 5-aminolevunlic acid (5-ALA) as a natural non-toxic photosensitizer involved in heme-biosynthetic pathway [32]. Although glycolytic inhibitors have been used previously in combination with PDT to induce cytotoxicity in MCF-7 cells [15], the novelty of our investigation stems from the use of Dichloroacetic acid (DCA), a mitochondrial-targeting non-toxic small molecule known to increase mitochondrial pyruvate uptake, in combination with 5-ALA-PDT. We provide evidence that DCA potentiates PDT-induced cytotoxicity in MCF-7 cells due to metabolic shift towards oxidative phosphorylation. Secondly, we demonstrate the release of ATP during PDT therapy with DCA indicating an involvement of immunogenic cell death mechanism. This is the first report on combinatorial treatment of DCA and PDT leading to cell growth inhibition and immunogenic cell death in MCF-7 cells which has potential to develop therapeutic application for cancer treatment.

## Materials and methods

### Reagents

Human breast adenocarcinoma MCF-7 cell line, DMEM media, Fetal Bovine Serum (FBS), penicillin, streptomycin and other cell culture reagents were obtained from the American Type Culture Collection (ATCC; Manassas, VA). Vybrant flow cytometry apoptosis assay kit (Catalog # V-13243) was obtained from Molecular Probes (Carlbad, CA). Dichloroacetic acid (DCA), 3-(4, 5-dimethylthiazol-2-yl)-2,5-diphenyltetrazolium bromide (MTT) reagent, dimethylsulfoxide (DMSO), 5’,5’,6’,6’-tetrachloro-1’,1’,3’,3’-iodide (JC-1) dye, 2’,7’-dichlorodihydrofluorescein diacetate (DCFH-DA) fluorescence dye, Trypan blue, 5-aminolevulinic acid (5-ALA) and ATP assay kit (Colorimetric/ Fluorometric, Cat. ab83355) were obtained from Sigma-Aldrich (St. Louis, MO).

### Cell culture

MCF-7 Cells were maintained in exponential growth phase in a complete medium containing DMEM, 10% FBS, penicillin (500 Units/ml) and streptomycin (500 Units/ml) as per manufacturer recommendation. All cells were grown in a humidified CO_2_ incubator set at 37˚C with 5% CO_2_ atmosphere. For subculturing, cells grown to 80% confluence were washed with PBS and then detached with Trypsin-EDTA buffer (0.05% Trypsin/0.53 mM EDTA in HBSS without sodium bicarbonate, calcium and magnesium) Subsequently, trypsin was removed by centrifugation and finally resuspended in fresh DMEM complete medium. Cell density was determined by counting the viable cells using a hemocytometer following staining with 0.4% Trypan blue dye.

### Cytotoxicity assay

The cytotoxic effects of DCA were assessed by MTT assay [33]. In brief, MCF-7 cells were seeded in 96-well plates (Costar, USA) at a density of 1.0–1.5x10^4^ cells per well. After overnight incubation at 37°C, cells were treated with various concentrations of DCA and incubated further for 24 h at 37°C with 5% CO_2_. Control wells received PBS alone. At the end of the incubation period of 24 h with DCA, the medium was discarded, washed twice followed by the addition of 10 μl MTT solution (5 mg/ml) to 90 μl culture medium containing no serum. The cells were incubated for an additional 4 h in the dark and thereafter, the medium was discarded, and the cells were lysed in100 μl of dimethylsulfoxide (DMSO) to dissolve the insoluble MTT formazan. The color thus developed was read at 570 nm in a microplate reader. The results were presented as % of the control values. All determinations were performed in triplicates, and each experiment was repeated at least three times.

### Photo-irradiation with laser light

**Fig 1:**
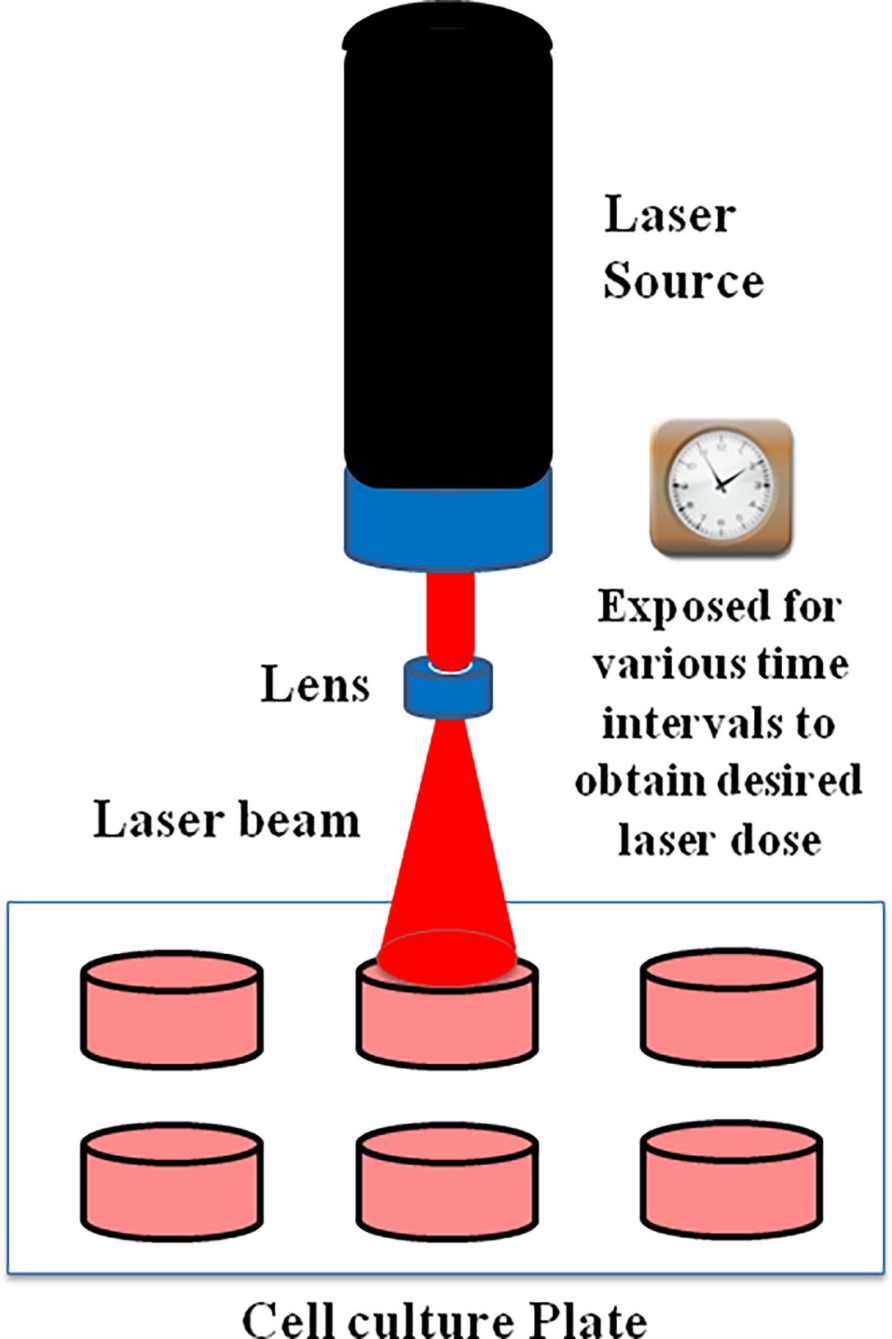
Schematic representation of laser irradiation. The beam from the He-Ne laser is directly focused on the cells grown in multi-well plates as a monolayer covering the entire surface area of the well. Cells were exposed for various time intervals in order to obtain indicated doses of laser irradiation.

For photo-irradiation experiments, MCF-7 cells were seeded in a clear 96-well microplate at a density of 1.5×10^4^ cells/well. The irradiation source was a He-Ne laser with an output power of 16 mW; the wavelength was 633 nm (Melles Griot-Covina; Rochester, NY) and this setup is shown in **Fig 1**. The spot size of the laser was maintained at 0.5 cm in diameter. Power measurements were made with the help of a meter (Instrumental Fiber Optics, Tempe, AZ, USA) and the exposure time was adjusted to obtain the desired doses of photo-irradiation in the range of 0–108 J/cm^2^. Control cells were not irradiated.

For photosensitization experiments, freshly prepared 5-ALA at 0.1 to 2.0 mM concentrations was used as a photosensitizer. Subsequent to treatments, cells were incubated at 37°C for 4 h followed by laser-irradiation at varying doses. At the end of the incubation, culture medium was discarded, washed twice with PBS and replaced with complete medium and the incubation was continued for another 24 h. To study the combined effects of PDT and DCA, the cells were first exposed to laser in the presence or absence of 5-ALA followed by DCA treatment for 24 h. The cell viability was then determined by MTT assay as described above.

### Flow cytometric analysis of apoptosis

Apoptosis in MCF-7 cells was assayed by flow cytometry as described earlier [34]. This assay detects changes in cell membrane permeability characteristics with YO-PRO-1 dye, a green-fluorescence nucleic acid stain that is permeant only to apoptotic cells but not to live cells. Necrotic cells are labeled with red-fluorescence propidium iodide. This assay detects three types of cell populations: (1) live cells show a low level of green fluorescence, (2) early apoptotic cells show an incrementally higher level of green fluorescence, (3) and late apoptotic (dead) cells show both red and green fluorescence. Using single-color stained cells, standard compensation was performed and cell debris was gated out [35, 36]. Briefly, 1 x 10^6^ cells/ml were incubated with 1μl of YO-PRO-I and 1 μl of PI for 30 min at 4^0^ C and were analyzed in a BD FACSCalibur (UAMS Core Facility for Flow cytometry) within 1 h. For each treatment condition, at least 10,000 cells were used and the data was analyzed by FlowJo software to detect different cell populations. YO-PRO-I based flow cytometry detects changes in plasma membrane phospholipid orientation, similar to annexin V-assay and these two methods are comparable. Data in the flow histogram is presented as percentages of early and late apoptotic cells in the total population. Bar graph shows total apoptotic cells in each treatment condition.

### Measurement of mitochondrial membrane potential (MMP)

For MMP measurements, MCF-7 cells were seeded in 96-well plates as described before and allowed to grow for 24 h. Cells were then incubated with varying concentrations of 5-ALA for 4 h. Excess 5-ALA was removed by a wash step followed by replacement with FBS and phenol red free DMEM. Cells were then irradiated with various doses of the He-Ne laser. Following laser irradiation, cells were washed twice with PBS and then treated with DCA for 24 h in complete DMEM medium. At the end of incubation, mitochondrial membrane potential was measured by JC-1 dye method as described earlier [37]. A change in red to green fluorescence ratio is considered a change in membrane potential. For red fluorescence, the excitation and emission wavelengths were set at 560 nm and 595 nm respectively, whereas for green fluorescence, the excitation and emission wavelengths were set at 485 am and 535 nm, respectively.

### Measurement of reactive oxygen species (ROS) production

For assessment of the production of intracellular ROS, cells were plated in the black clear bottom 96-well plates at a density of 1.5 x 10^4^ cells/well and treated with 5-ALA for 4 h as described above. After washing the cells with PBS, cells were irradiated with the laser as detailed earlier. Finally, the cells were treated with DCA for an additional 24 h. At the end of the incubation period, ROS was detected using 2’, 7’-dihydrochloroflurorescein acetate (DCFH-DA) as described earlier [38]. Briefly, DCFH-DA was added to the medium at a final concentration of 10 μM and cells were incubated for 90 min in dark. Subsequently, cells were washed twice and resuspended in 100 μl of PBS. DCFH-DA staining intensity was measured using a fluorescence microplate reader (SYNERGY H4, Bio Tek, hybrid technology) at excitation and emission wavelengths of 485 nm and 535 nm, respectively.

### ATP release assay

For ATP release assay, MCF-7 cells were seeded in 12-well plates at a density of 1 x 10^5^ cells/well and incubated at 37°C for 24 h. Cells were then treated with 1.0 mM 5-ALA for 4 h and exposed to laser light (108 J/cm^2^) followed by treatment with 10 mM DCA as described above. ATP released in cell culture medium was measured using a commercially available kit. Briefly, cell lysates prepared in 100 μl ATP Assay buffer and the cell debris were removed by centrifugation at 13,000 g for 5 min at 4°C. Samples were then deproteinized with 1 M perchloric acid and neutralized with potassium hydroxide. The clear supernatants were obtained following centrifugation at 13,000 g for 5 min at 4°C to remove any insoluble particles for ATP determination. An aliquot of 50 μl of the sample was mixed with 50 μl of ATP reaction mix and the relative fluorescence units were determined at excitation and emission wavelengths of 535 nm and 587 nm, respectively.

### Statistical analysis

All calculations were performed using GraphPad prism v 6.0 (San Diego, CA). Each experiment was repeated at least three times. All data that show error bars are presented as mean ± SE unless otherwise mentioned. The significance of difference in the mean values was determined using two-tailed Student’s ‘t’ test or by ANOVA using prism software. The p-value *p≤ 0.05; **p≤0.01, were taken as the value with significant differences as compared with their controls.

## Results

### DCA induces cell growth inhibition in MCF-7 Cells

**Fig 2:**
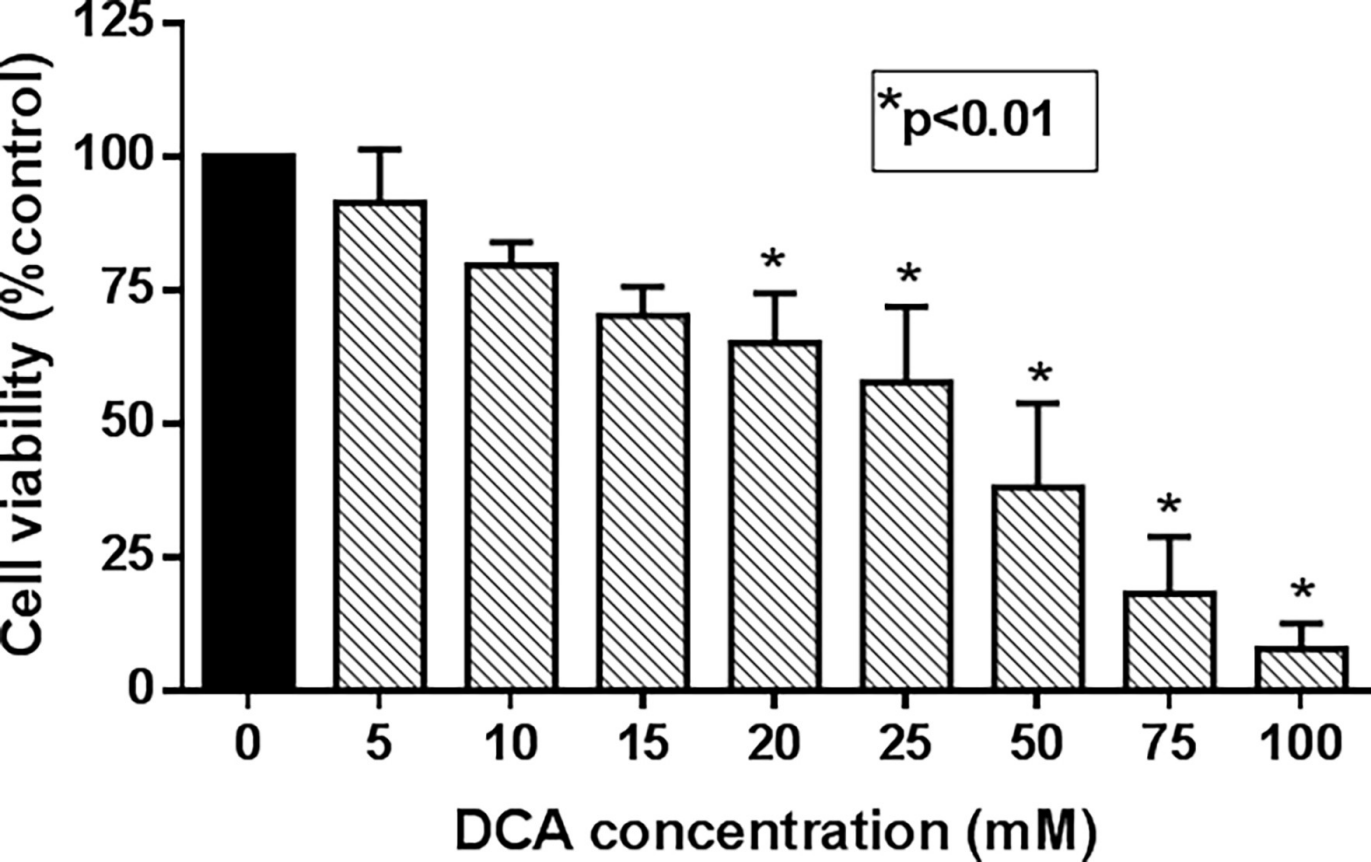
Effect of DCA on cell viability. MCF-7 cells were plated at a density of 1.5x10^4^ cells per well in 96-well plates and grown for 24 h. Cell were then treated with indicated doses of DCA for 24 h and the cell viability was determined by MTT assay as described in “Materials and Methods” section. Data shown are mean values ± standard error (SE) from 3 to 4 independent experiments, each performed in triplicate. Asterisk shows values significantly different from control. *P ≤ 0.01.

We have previously shown that DCA induces cell death in breast cancer adenocarcinoma MCF-7 cell line via mitochondrial-dependent ROS production. We then hypothesized that DCA might potentiate and produce synergistic effect on MCF-7 cells when combined with He-Ne laser based photodynamic therapy (PDT). Cell growth inhibition in MCF-7 cells was performed using MTT assay. As shown in **Fig 2,** DCA alone affected cell growth inhibition in a dose-dependent manner during 24 h treatment. The cytotoxic effects of DCA were more significant at higher doses of DCA (>20 mM). For combinatorial studies with DCA and PDT, we chose a non-cytotoxic dose of DCA (10 mM) in the follow up experiments.

### Low dose DCA and laser irradiation cause minimal effect on cell viability

**Fig 3:**
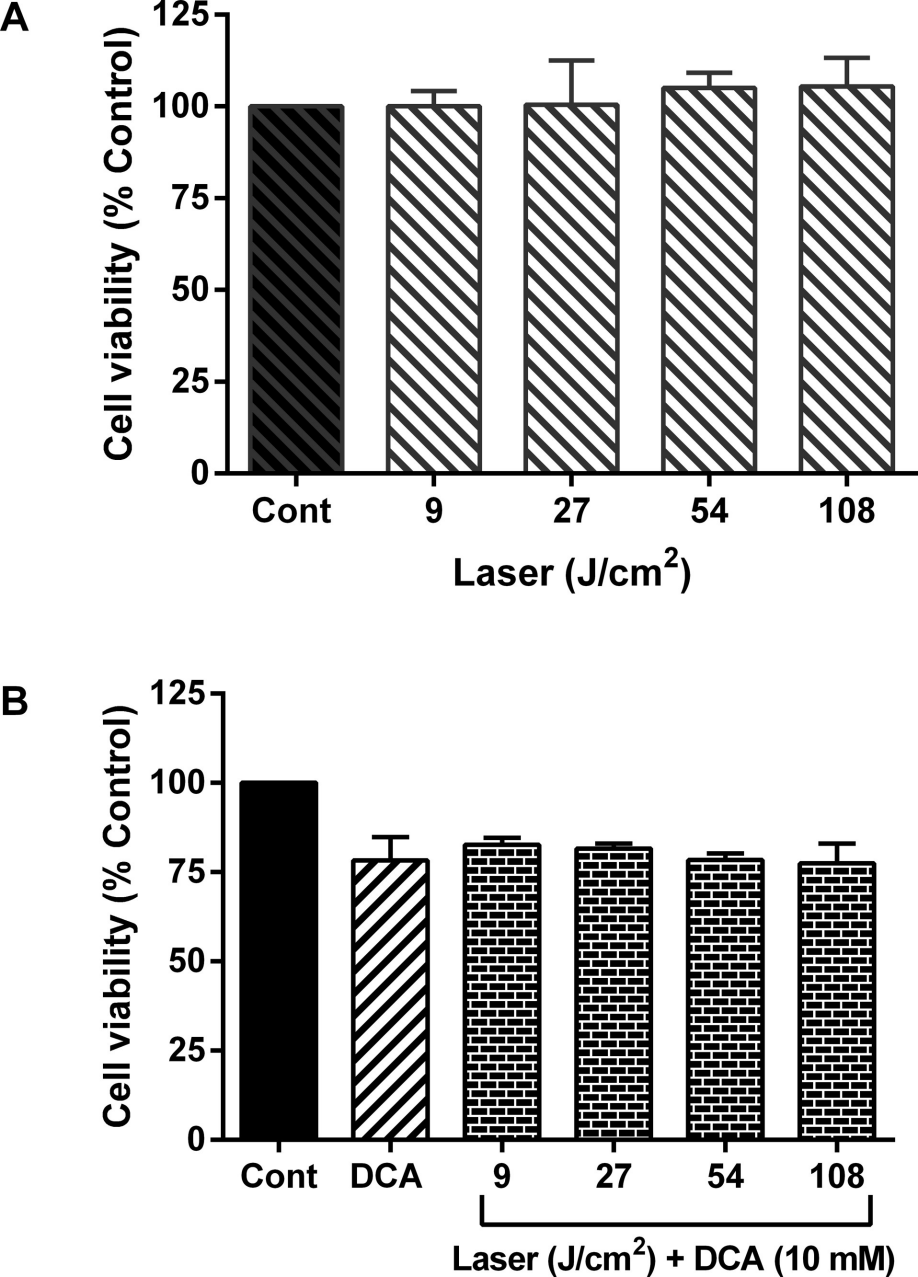
Combined effect of laser-irradiation and DCA on cell viability. MCF-7 cells were cultured in 96-well plates as above in Fig 2. Cells were irradiated with indicated doses of laser light following treatment with (B) or without (A) 10 mM DCA. DCA alone without laser irradiation was also included as a control. Data shown are mean values ± SE from three independent experiments, each performed in triplicate.

The effects of He-Ne laser up to a maximum dose of 108 J/cm^2^ and 10 mM DCA were first tested alone and in combination with DCA to examine their effects on cell viability of MCF-7 cells. As shown in **Fig 3,** the cell viability of MCF-7 was not affected significantly by either treatment with He-Ne laser or DCA alone or in combination of the two where cells were treated with DCA following exposure to laser. These results suggest that no significant cytotoxicity associated with the laser and DCA at the dose regimens employed in this study.

### Optimal dosing of photodynamic therapy following photosensitization with 5-ALA

**Fig 4:**
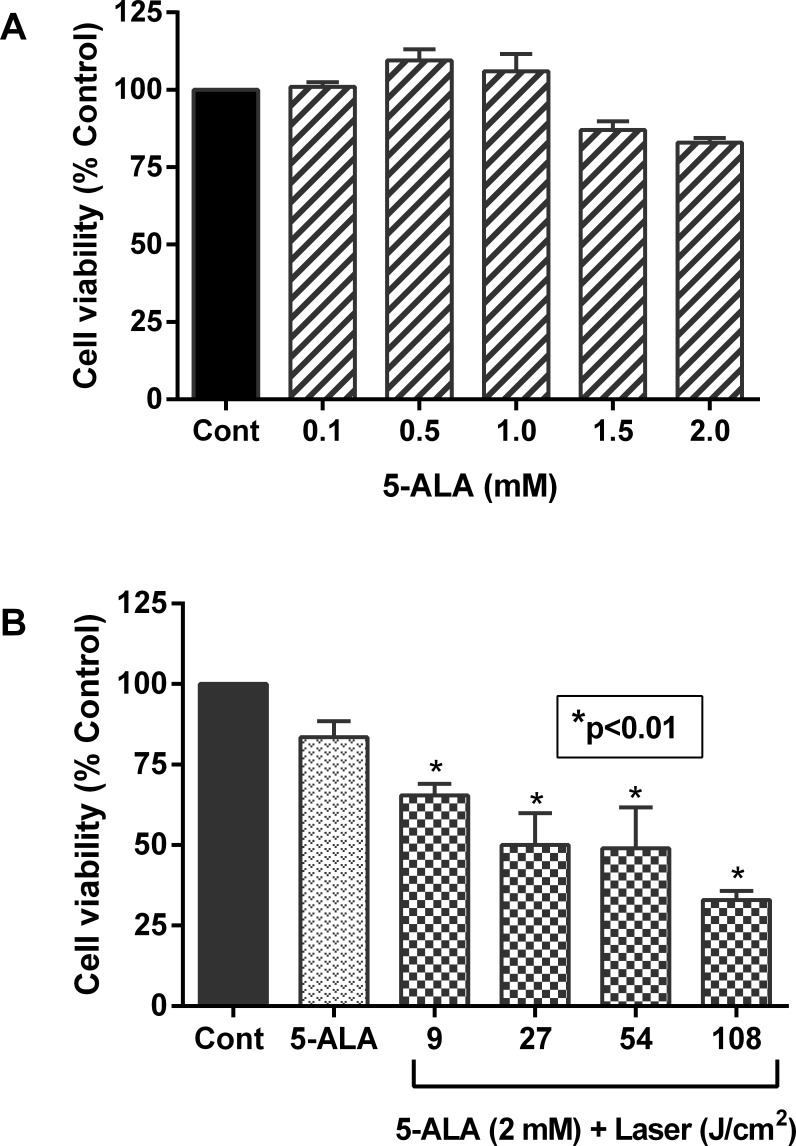
Effect of 5-ALA and laser-irradiation on cell viability. MCF-7 cells were cultured in 96-well plates as above in **Fig 3** and then treated with indicated concentrations of 5-ALA alone as a photosensitizer for 4 h (**A**). For combined treatment (**B**), cells were first treated with 1.0 mM 5-ALA and then exposed to indicated doses of laser-irradiation. Controls without any treatment and only with 5-ALA were also included. Cell viability was determined by MTT assay as described in “Materials and Methods” section. Data shown are mean values ± SE from three independent experiments, each performed in triplicate. Asterisk shows values significantly different from control. *P ≤ 0.01.

In order to develop an effective photodynamic therapy approach with laser irradiation, we used 5-aminoluvelinic acid (5-ALA) as a photosensitizer in this study. As shown in **Fig 4A**, 5-ALA itself did not cause any significant changes in cell viability of MCF-7 cells even at 2.0 mM, the maximum dose used in this study. However, when the cells were irradiated with the laser light following treatment with 2.0 mM 5-ALA, a marked decrease in cell viability was observed. These changes were dependent upon the laser beam strength employed (**Fig 4B**). These results suggest that 5-ALA is a safe photosensitizer to develop a meaningful PDT therapy for breast cancer.

### Dose-dependent sensitizing effect of 5-ALA on DCA-mediated cell growth inhibition

**Fig 5:**
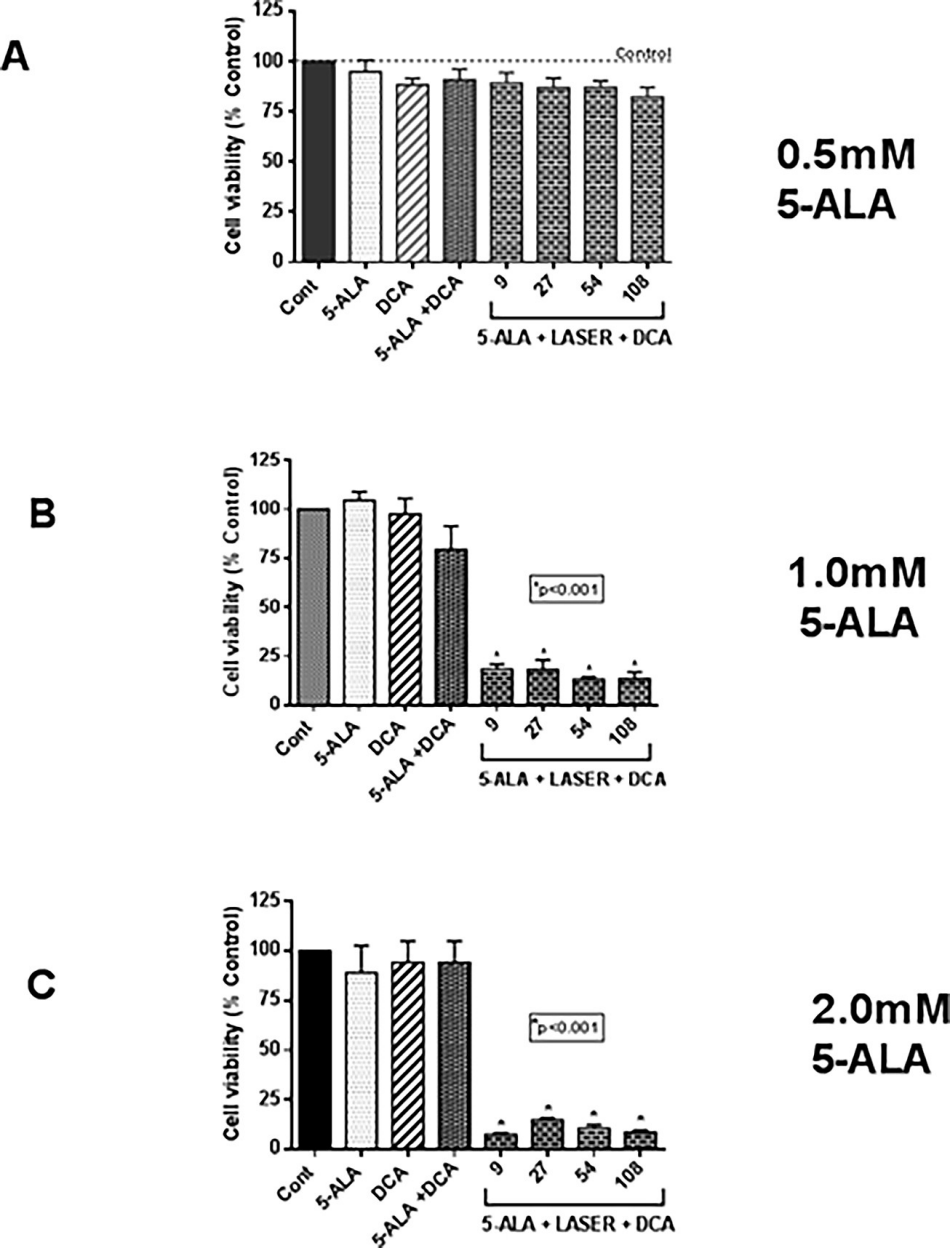
Dose-dependent sensitizing effect of 5-ALA on laser-irradiation and DCA mediated effects on cell viability. MCF-7 cells were cultured in 96-well plates as above in **Fig 3** and then treated with 5-ALA (**A**, 0.5 mM; **B**, 1.0 mM; **C**, 2.0 mM) for 4 h followed by indicated doses of laser-irradiation. Cells were then treated with 10 mM DCA. Controls with 5-ALA and DCA alone and in combination without laser irradiation or with any treatment were also included. Cell viability was determined by MTT assay as described in “Materials and Methods” section. Data shown are mean values ± SE from three independent experiments, each performed in triplicate. Asterisk shows values significantly different from control. *P ≤ 0.01.

We next studied whether 5-ALA can sensitize the cytotoxic effects of DCA on MCF-7 cells following the combined treatment with 5-ALA and laser irradiation based PDT (5-ALA-PDT). In a series of experiments, we found the optimal dose at which 5-ALA effected the highest cell growth inhibition. Similarly, we kept the concentration of DCA to 10 mM and the laser beam strength constant. As shown in **Fig 5**, 0.5 mM concentration of 5-ALA caused no significant effect on cell growth (**Fig 5A**). However, at doses with 1.0 mM and 2.0 mM, 5-ALA caused synergistic effects (**Fig 5B and Fig 5C)** suggesting 5-ALA sensitizing effect was dose-dependent. However, beyond 1.0 mM concentration, there was no significant increase in cell growth inhibition by 5-ALA. We therefore used 1.0 mM 5-ALA in follow up experiments to investigate the cell death mechanism.

### DCA promotes 5-ALA-PDT-induced apoptosis

**Fig 6:**
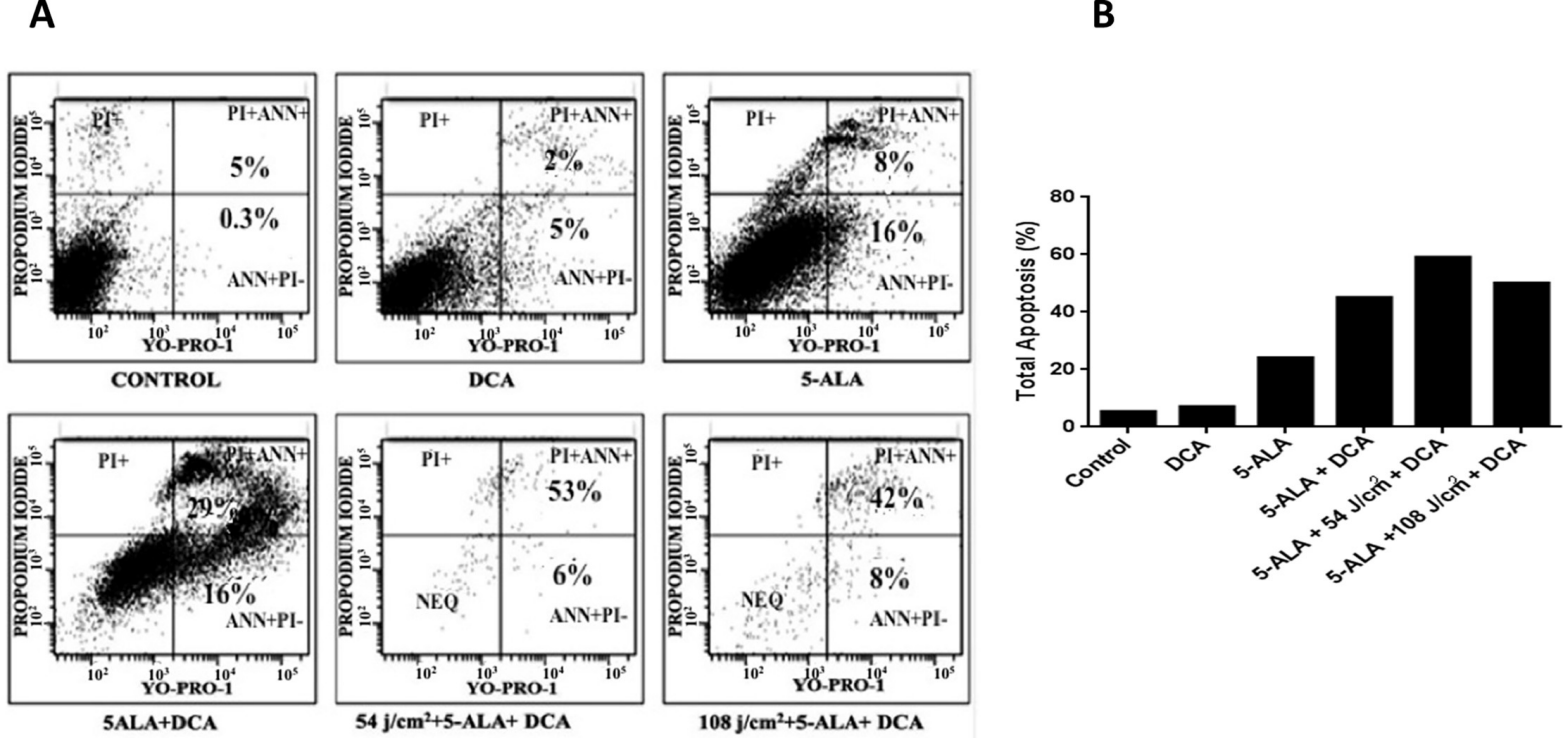
Flow cytometry analysis of apoptotic cells. MCF-7 cells were cultured in 6-well plates and treated as described in Materials and Methods section. Harvested cells (1 x 10^**6**^ cells/ml) were labeled with the Vybrant apoptosis assay kit reagents (Yo-Pro-1 and propidium iodide) and analyzed in a BD FacsCalibur flow cytometer. Fig 6A: Early apoptotic cells were identified by Yo-Pro-1 staining (green fluorescence, right lower quadrant), whereas late apoptotic events were identified by dual staining of Yo-Pro-1 and PI (red fluorescence, right upper quadrant). Results show a marked increase in early and late apoptotic events in the combined treatment with DCA and 5-ALA-laser-irradiation. Shown is a representative histogram out of three independent experiments performed with similar results. Fig 6B: Bar graph showing total apoptotic cells with various treatments. Data from flow cytometry experiments (Fig 6A) were represented here in the form of bar graphs. It is evident that DCA + PDT enhance cell death by apoptosis.

Next, we asked whether the cell growth inhibition observed during 5-ALA-PDT experiments were due to apoptotic cell death. Using a commercially available kit (YO-PRO-I), early and late apoptotic cells were analyzed. As shown in **Fig 6A**, early apoptotic cells stained with YO-PRO-I showed green fluorescence for apoptotic cells (lower and upper right quadrants) and late apoptotic cells stained with propidium Iodide show red fluorescence for necrotic cells (upper left quadrants). Double staining with YO-PRO-I and PI is indicative of late apoptotic cells in the culture. As compared to controls, PDT + DCA treatment resulted in the highest apoptotic cell death (**Fig 6A & 6B**). When the cells were exposed to 1.0 mM 5-ALA and laser light (54 and 108 J/cm^2^) followed by treatment with 10 mM DCA, there was a significant loss of cells for flow cytometric analysis perhaps due to necrosis (Fig 6A). The lower number of apoptotic cells seen in the histogram is indicative of the remaining intact cells in the culture. These results are in agreement with our cell viability data shown earlier. Collectively, our results clearly demonstrate that DCA enhances 5-ALA-PDT mediated cell death by apoptosis.

### Combinatorial treatment of DCA and 5-ALA-PDT induces mitochondrial damage

**Fig 7:**
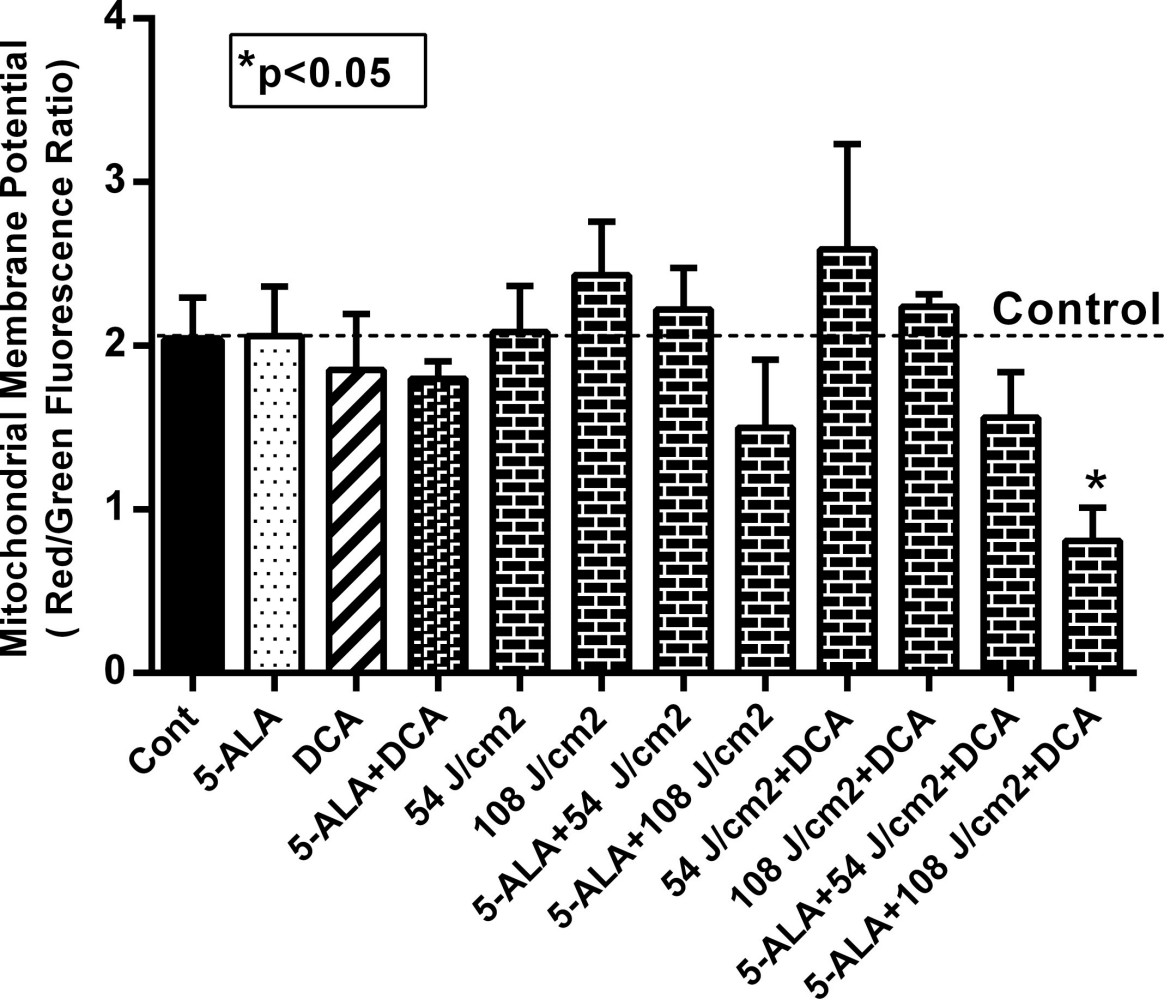
Effect of DCA on 5-ALA and Laser-irradiation on mitochondrial membrane potential. MCF-7 cells were seeded at a density of 2.0–2.5 x 10^4^ cells/well in 96-well plate and grown as described above. Cells were treated with 1.0 mM 5-ALA, 10 mM DCA and 54 and 108 J/cm2 laser doses alone or in combination as described above. The cells were then stained with JC-1 (10 μM) for 20 min at 37°C in the dark. The ratio of red to green fluorescence was acquired subsequently with a fluorescence microplate reader. Data shown are mean values ± SE from three independent experiments, each performed in triplicate. Asterisk shows value significantly different from control. *P ≤ 0.01.

**Fig 8:**
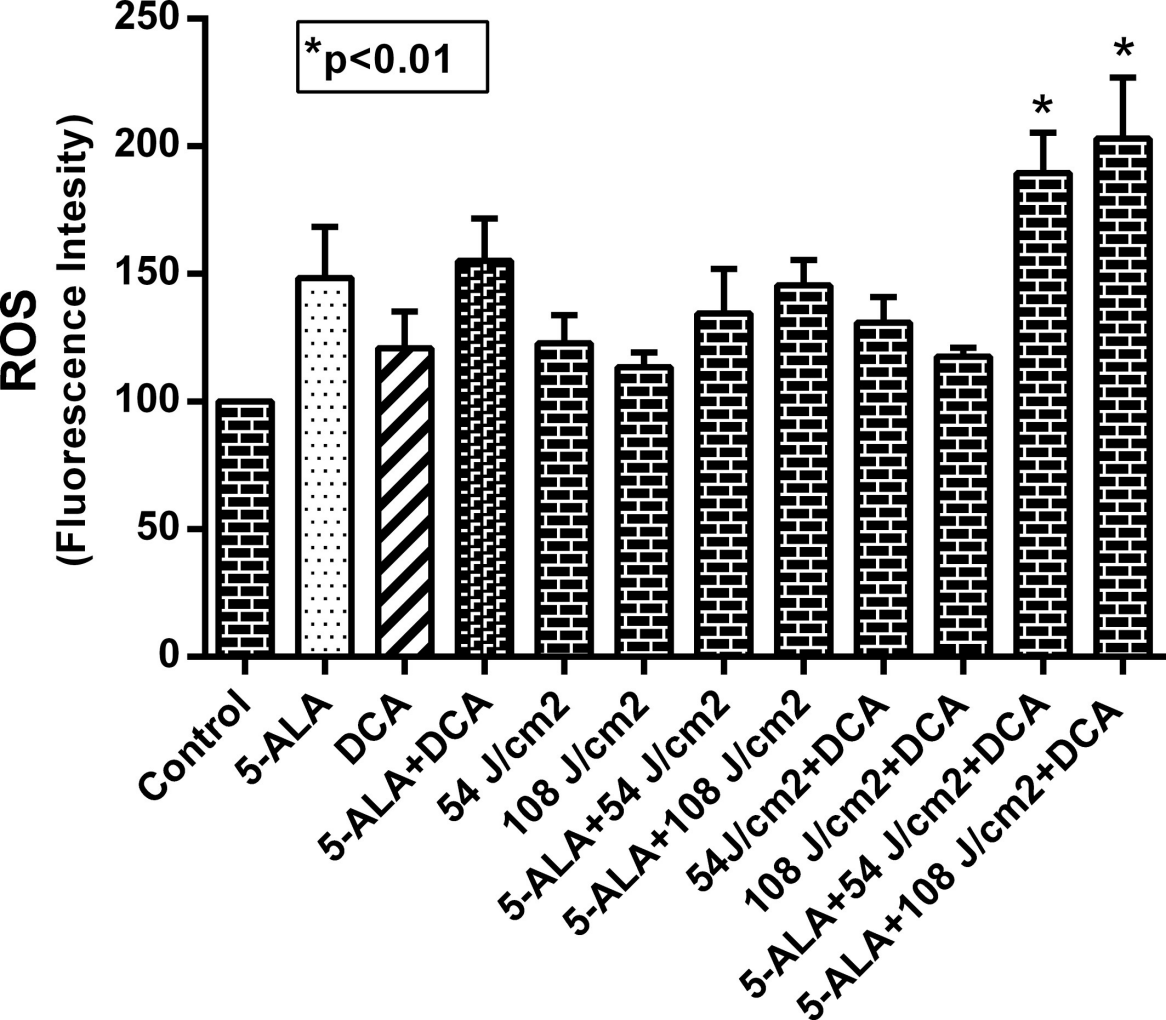
Effect of DCA on 5-ALA and laser-irradiation on ROS production. MCF-7 cells were plated in the black clear bottom 96-well plates at a density of 1.0–1.5×10^4^ cells/well and grown as described in Materials and Methods section. The cells were treated with 1 mM 5-ALA, 10 mM DCA and indicated does of laser alone or in combination. The cells were then stained with DCFH-DA and fluorescence intensity was determined. Data shown are mean values ± SE from three independent experiments, each performed in triplicate. Asterisk shows value significantly different from control. *P ≤ 0.01.

Earlier studies have shown that DCA and PDT treatment led to increased ROS production and oxidative stress in various experimental models [26, 39, 40]. Since mitochondria is involved in excess ROS production, we treated MCF-7 cells with 10 mM DCA and 1.0 mM 5-ALA, and two different laser beam strengths (54 and 108 J/cm^2^) to determine ROS associated oxidative stress. Our results with mitochondrial membrane potential (Δψm) assessment in MCF-7 cells showed that none of the treatments either alone or in combination resulted in any significant change in MMP (Δψm) as compared to controls, except when cells were subjected to 108 J/cm2 dose of laser-irradiation (5-ALA-PDT). A significant drop (50% reduction to controls) in Δψm was observed as shown in **Fig 7**. These results suggest that the beneficial effects of combinatorial treatments involve mitochondrial integrity and are dependent on laser irradiation at 108 J/cm2. We next investigated DCFH-DA fluorescence as a measure of ROS production during DCA based photodynamic therapy under various experimental conditions. Similar to the results shown in **Fig 7**, ROS production (**Fig 8**) paralleled mitochondrial membrane potential suggesting oxidative stress played a vital role in mitochondrial dysfunction. Thus, it is likely that decreased cell viability and increased apoptosis reported earlier (**Figs 5 and 6**) could be due to increased ROS and mitochondrial dysfunction.

### Immunogenic cell death by DCA during photodynamic therapy

**Fig 9:**
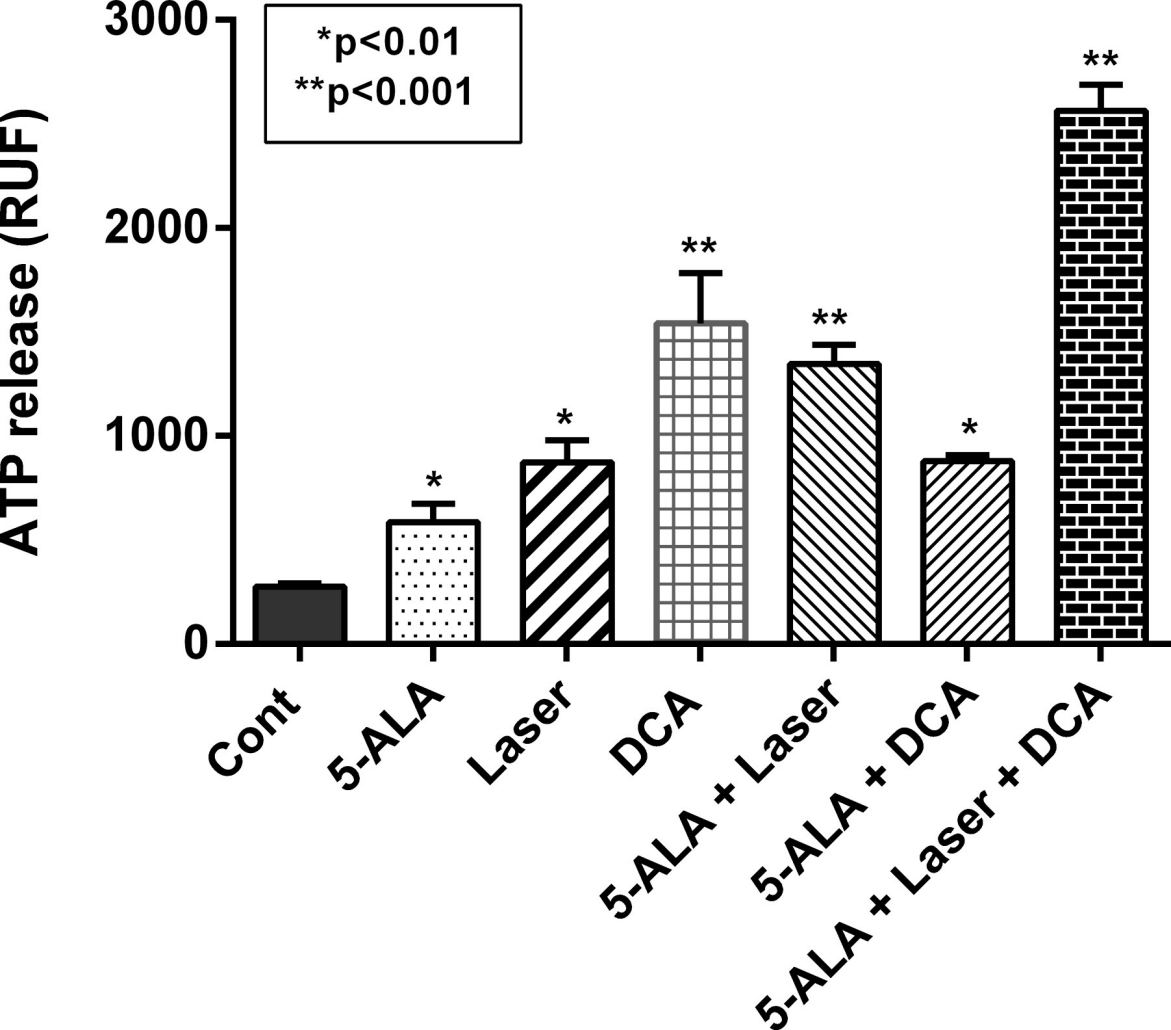
ATP Release in response to DCA, 5-ALA and laser-irradiation. MCF-7 cells were seeded at a density of 1x 10^5^ cells/well in 12-well plates and grown as described above. Cells were treated with 1.0 mM 5-ALA, 108 J/cm2 laser dose and 10 mM DCA alone or in combination as indicated. The release of ATP was measured by relative fluorescence. Data shown are mean values ± SE from three independent experiments, each performed in triplicate. Asterisk shows values significantly different from control. *P ≤ 0.01, **P ≤ 0.001.

Accumulating literature strongly suggest a beneficial liaison between radiotherapy and immunotherapy [28]. Importantly, local radiation produces systemic, immune mediated anti-tumor and, potentially antimetastatic effect via release of DNA, neoantigens and ICD [28, 41, 42]. In this study, we investigated whether our approach of DCA treatment following 5-ALA and laser-irradiation (5ALA-PDT) is linked to ICD. For this, we measured ATP release as one of the DAMPS molecules in response to various treatment conditions. As shown in **Fig 9**, treatment of MCF-7 cells with 1.0 mM 5-ALA, 108 J/cm^2^ laser-irradiation and 10 mM DCA alone and in combination caused a significant release of ATP. These doses of treatments, as determined in cell viability studies, were nontoxic. The release of ATP was most prominent when cells were treated with DCA following treatment with 5-ALA and laser-irradiation (**Fig 9**). Thus, the ATP release paralleled mitochondrial damage, ROS production and cell death indicating that these molecular events are inter-related culminating into immunogenic cell death.

## Discussion

The purpose of this study was to investigate the potential role of DCA in reprogramming cellular metabolism of MCF-7 breast cancer cells in the presence of radiosensitization by photodynamic therapy. Garg *et al* and others [28, 31, 43, 44] have demonstrated that PDT provides all the benefits of the localized ionizing radiation therapy in a ROS-dependent manner. The data included in this report demonstrate that combinatorial therapy involving DCA and laser based PDT has an additive effect on MCF-7 cells resulting in decreased cell growth and enhanced apoptotic cell death. To the best of our knowledge, this is the first report demonstrating induction of immunogenic cell death with the assistance of radiosensitization and DCA treatment in a breast cancer cell line. Recent breakthroughs in immunotherapy have shown that eliciting immune responses against multiple types of cancer can lead to a wide range of benefits including complete regression of metastatic disease in some cases [45]. Furthermore, encouraging results from combination with PDI and PDL1 inhibitors with cytotoxic chemotherapy and/or radiation therapy has accelerated a renewed interest in the use of combination therapies in oncology practice. Recently, Verbrugge et al [46] have demonstrated that radiotherapy can neutralize the inhibitory role of immune checkpoint pathways, thus suggesting that radiotherapy may benefit from coincident or subsequent immunotherapy [8, 28, 47, 48].

Radiotherapy has been a major component of cancer treatment, with over 50% of cancer patients receiving radiation during the course of their disease. As a monotherapy, radiation is widely known to induce tumor cell death through DNA damage and by promoting expression of Fas and MHC class I antigens on tumor cells [28]. In recent years, various combination therapies involving surgery, radiation, chemotherapy, and immunotherapy are favored over monotherapies since cancer is a complex disease affecting multiple signaling pathways [28, 49]. Along these lines, radiation therapy is the most favored tool since it is inclusive of both immuno-enhancing and free radical generating therapy. Furthermore, the “abscopal effect” reported for radiotherapy (RT) wherein, the localized RT results in immune-mediated tumor regression in disease sites well outside of the radiation field [28, 41]. These ancillary and largely unappreciated immunologic effects are now being recognized and intensely researched to better understand the changes within the tumor microenvironment.

Both chemotherapy and radiation therapy have been causally linked to induction of apoptosis in various cancer models [50]. While apoptosis leads to a spectrum of immunological consequences, ICD is more specific and potent way in activating tumor-specific immunity [28, 50]. As a consequence of ICD, damage-associated molecular patterns (DAMPs), such as ATP, uric acid and high mobility group protein Box1 (HMGB1), from tumor cells and by the translocation of calreticulin, an ER- associated chaperone, to the tumor cell surface are released and amplify immune response [51, 52]. Similarly, extracellular ATP is an important chemo-attractant for immune cells and plays a major role in the activation of dendritic cells [28, 50]. Taken together, these immunological determinants activate both innate and adaptive immune responses that ultimately enhance anti-tumor responses.

**Fig 10:**
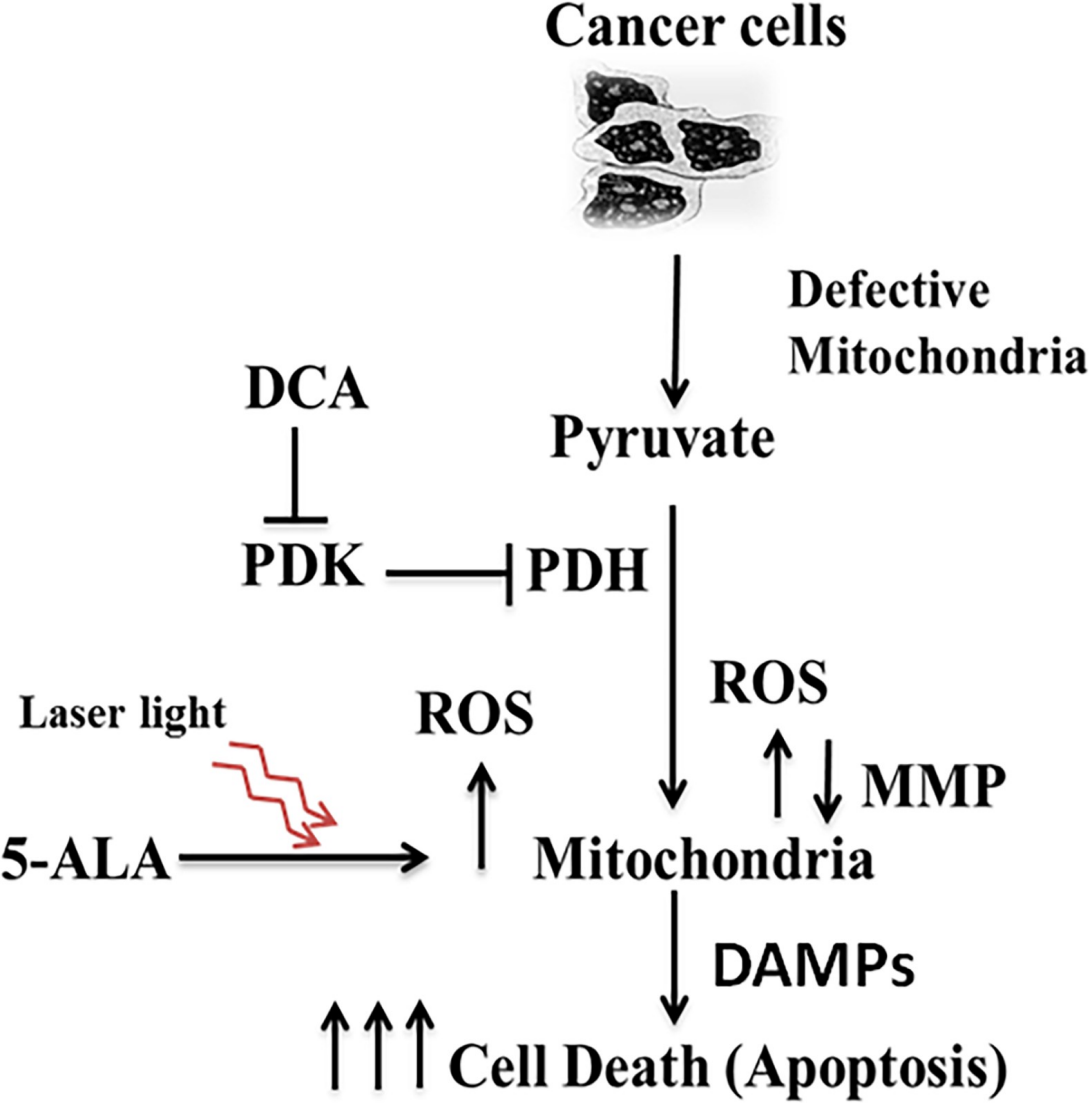
Proposed mechanism of apoptosis by PDT and DCA. Possible mechanism of apoptosis in MCF-7 cancer cells is via immunogenic cell death signaling pathway involving mitochondrial damage, ROS production and ATP release (DAMPs).

The biologic premise behind the combinatorial therapy of DCA and radiotherapy is that the tumor-antigen release together with augmented Warburg effect facilitates enhanced oxidative stress and immunogenic cell death. Till recently, chemotherapy drugs prescribed for cancer patients were considered cytotoxic as well as immunosuppressive [49]. However, recent data indicate that some of these drugs are not only cytotoxic, but also facilitate an adaptive immune response against the tumor that the patient is suffering from. Most solid tumors including breast cancer switch to glycolytic metabolism even though oxygen is sufficiently available for oxidative phosphorylation to produce needed cellular energy [9]. Targeting this abnormal phenomenon has paved a way for developing novel cancer therapeutic strategies. DCA is known to bring about such a metabolic shift [16, 17] by inhibiting pyruvate dehydrogenase kinase (PDK), thus activating pyruvate dehydrogenase (PDH) which enables mitochondrial pyruvate uptake. DCA has also been shown as an effective anticancer agent [18, 53, 54], which enhances therapeutic benefit in certain types of cancer cells [55]. In the present study, we have examined the effectiveness of DCA by combining it with 5-ALA as a photosensitizer and He-Ne laser as a mild radiation source to achieve apoptosis in MCF-7 human breast cancer cells. We have demonstrated that DCA has robust apoptotic effects when combined with PDT. Further, our results provide a mechanistic explanation of this treatment whereby a combination of elevated ROS, mitochondrial damage and release of ATP lead to immunogenic cell death pathway (**Fig 10**).

Photodynamic therapy has been shown to be beneficial in treating various solid tumors because of the lack of side effects that ionizing radiation known to produce [29, 30]. PDT can be combined with other cytotoxic cancer drugs, but at significantly lower doses to minimize deleterious side effects, yet maximize its effectiveness. Golding et al (2013) has demonstrated that glycolytic inhibitors such as 2-deoxyglucose or lonidamine potentiate 5-ALA based PDT by inhibiting ATP production [15]. This study overlooked ATP leakage outside the tumor cells and associated immunological consequences of ATP related signaling. In our study, we have shown that DCA potentiates PDT by two possible mechanisms, firstly by targeting oxidative phosphorylation and secondly by immunogenic cell death and apoptosis. Additionally, we argue that ATP acts as a DAMP molecule in light of large body of literature available now. To our knowledge, this is the first report showing DCA together with PDT on immunogenic cell death. In our study, we have used low doses of He-Ne laser light with specific wavelength of 633 nm as a mild radiation source. This wavelength of laser light specifically strikes certain photosensitizer molecules and energizes them to produce ROS and cause oxidative damage [27]. For this purpose, we chose 5-aminolevunlic acid (5-ALA) as a non-toxic photosensitizer known to be involved in heme biosynthetic pathway [32]. 5-ALA preferentially enters cancerous cells perhaps due to their genetic variability and hyper proliferative biochemical nature [56–58]. Being a natural precursor molecule of heme biosynthesis, 5-ALA is non-toxic to the cells. 5-ALA produces protoporphirin IX (PpIX) as its first metabolite in heme synthesis which is accumulated in large quantities within the mitochondria. PpIX is highly sensitive to photo-irradiation and upon laser exposure it produces excessive ROS which amounts to mitochondrial damage. Thus, 5-ALA is a preferred photosensitizer in PDT. However, the degree of effectiveness of 5-ALA based PDT is highly dependent upon the nature of cancer cell type [32, 59].

In our combined approach of DCA mediated PDT treatment strategy, we have first optimized concentrations of DCA, 5-ALA and doses of laser exposure to determine their individual non-toxic values in a MTT based cytotoxicity assay. The doses of DCA and 5-ALA used in our combination treatment are very safe and non-toxic [53, 54, 60]. Similarly, laser doses used in our study also did not have any deleterious effect as a single agent treatment. Mechanistically, both DCA and PDT enhanced ROS production and decreased mitochondrial membrane integrity paving the way for increased apoptosis [60].

The type of danger signals in the form of DAMPs released during DCA plus PDT in our cell culture system is still for speculation. However, our preliminary data on ATP shows that this molecule is significantly elevated suggesting a vital role for ICD in our cell culture model (**Fig 10**).

## Conclusion

Tumor cells are notoriously non-immunogenic and acquire metabolic transformation and therapy resistance, the properties that enable them to evade the immuno-surveillance system. The data included here have identified a role for DCA and PDT in the induction of apoptotic and immunogenic cell deaths. Our data indicate that DCA can be combined with other treatment agents, such as those used in photodynamic therapy, to induce cytotoxicity or apoptosis primarily in cancerous cells. Our studies also demonstrate a safe use of this combination approach to target aggressive cancerous cells. Since DCA has been used in humans and clinical trials are still underway, it is safe to conduct further studies of its use in laser therapy. In the future, testing DCA with the photosensitizer and laser-irradiation in an *in vivo* animal model of tumor xenograft will be a meaningful step in the right direction to further realize its potential as a successful drug for cancer treatment.
